# Recovering stimulus locations using populations of eye-position modulated neurons in dorsal and ventral visual streams of non-human primates

**DOI:** 10.3389/fnint.2014.00028

**Published:** 2014-03-28

**Authors:** Anne B. Sereno, Margaret E. Sereno, Sidney R. Lehky

**Affiliations:** ^1^Department of Neurobiology and Anatomy, University of Texas Health Science Center at HoustonHouston, TX, USA; ^2^Department of Psychology, University of OregonEugene, OR, USA; ^3^Computational Neurobiology Laboratory, The Salk Institute for Biological StudiesLa Jolla, CA, USA

**Keywords:** monkey, spatial vision, active vision, population coding, multidimensional scaling

## Abstract

We recorded visual responses while monkeys fixated the same target at different gaze angles, both dorsally (lateral intraparietal cortex, LIP) and ventrally (anterior inferotemporal cortex, AIT). While eye-position modulations occurred in both areas, they were both more frequent and stronger in LIP neurons. We used an intrinsic population decoding technique, multidimensional scaling (MDS), to recover eye positions, equivalent to recovering fixated target locations. We report that eye-position based visual space in LIP was more accurate (i.e., metric). Nevertheless, the AIT spatial representation remained largely topologically correct, perhaps indicative of a categorical spatial representation (i.e., a qualitative description such as “left of” or “above” as opposed to a quantitative, metrically precise description). Additionally, we developed a simple neural model of eye position signals and illustrate that differences in single cell characteristics can influence the ability to recover target position in a population of cells. We demonstrate for the first time that the ventral stream contains sufficient information for constructing an eye-position based spatial representation. Furthermore we demonstrate, in dorsal and ventral streams as well as modeling, that target locations can be extracted directly from eye position signals in cortical visual responses without computing coordinate transforms of visual space.

## Introduction

In this study we compare spatial representations in a dorsal area, lateral intraparietal cortex (LIP), and a ventral area, anterior inferotemporal cortex (AIT). We recorded from LIP and AIT because both are high-level visual areas in the dorsal and ventral streams respectively, where differences between dorsal and ventral processing are likely to be most salient. While LIP is generally associated with representations of space (Colby and Duhamel, 1996), AIT has in the past been commonly associated with the representation of complex shapes for object recognition (Tanaka, 1996) rather than space.

We shall be concerned solely with spatial information derived from modulations in neural activity caused by changes in eye position (gaze angle), as occurs when the eyes fixate a particular stimulus presented at different locations. The retinal position of the stimulus is constant, but eye position changes. Conceptually this eye-position based space is quite different from the most commonly studied situation, retinotopic-based space. In measuring retinotopic-based space, eye position is constant but the retinal location of the stimulus changes. We have previously conducted a study of retinotopic-based space (Sereno and Lehky, 2011), which showed that a representation of retinotopic space does indeed exist in AIT, as well as reported on dorsal/ventral differences in retinotopic spatial representations. Here we present an analogous study of eye-position based space.

Eye-position modulations of visual cortical responses have long been established in the dorsal stream, notably in area 7a and LIP (Andersen and Mountcastle, 1983; Andersen et al., 1985, 1990). More recently, eye position has also been demonstrated to modulate neural responses in the ventral visual stream, including cells in V4 (Bremmer, 2000; Rosenbluth and Allman, 2002), inferotemporal cortex (Lehky et al., 2008), as well as hippocampus and parahippocampus (Ringo et al., 1994).

Responses of a cell as a function of eye position are often described as the gain field for that cell. When mapping out a gain field, the same stimulus is always viewed at the same retinal location; just the gaze angle (i.e., angle of the eye in the orbit) changes. For some gaze angles, firing rates can be substantially higher than other gaze angles, even though there is no change in the retinal stimulus. Previous work has often approximated gain fields as being planar (e.g., Andersen et al., 1990; Bremmer, 2000). If firing rates are plotted in the third dimension as a function of the x and y coordinates of eye position, then these gain fields can be visualized as tilted 3D planes.

Here we extract eye position from a population of cells having a diversity of gain fields (that is, each cell is modulated by eye position in a different way). If a stimulus is fixated, then decoding eye position is equivalent to decoding stimulus position. By localizing stimuli in this way, we adopt a completely different approach than what has been used in the past. In the past, eye-position modulations or gain fields have often been interpreted as a mechanism for producing spatial coordinate transformations between a retinotopic frame of reference and a head-centered frame of reference (Zipser and Andersen, 1988; Andersen et al., 1993; Snyder, 2000; Salinas and Abbott, 2001; Lehky et al., 2008). In contrast, here we use gain fields (i.e., eye position signals) alone to localize stimuli directly, without any changes in coordinate systems defining the physical space in which neurophysiological signals can be interpreted.

Eye positions are extracted from our data using population decoding methods. We have had recent success with a fundamentally different approach (namely, multidimensional scaling, MDS) to population decoding than is most commonly used (reviewed in Lehky et al., 2013). In addition to our previous work on retinotopic space (Lehky and Sereno, 2011; Sereno and Lehky, 2011), MDS methods have been used to decode visual shape (Rueckl et al., 1989; Young and Yamane, 1992; Rolls and Tovée, 1995; Murata et al., 2000; Op de Beeck et al., 2001; Kayaert et al., 2005; Kiani et al., 2007; Lehky and Sereno, 2007). MDS is an example of what we call an *intrinsic* method for population decoding, in contrast to such popular methods as weighted peak averaging or Bayesian estimation, which are *extrinsic* decoding methods. We have extensively discussed these two approaches, as well as reviewed the representational advantages that intrinsic (as opposed to extrinsic) approaches offer (Lehky et al., 2013); see also Kriegeskorte and Kreiman (2011), whose multivariate approach to population coding is similar to what we call intrinsic coding.

Using this same intrinsic approach we previously used for retinotopic visual space, we show for the first time that stimulus locations can be recovered solely from eye-position modulations; see (Sereno, 2011; Sereno and Lehky, 2012) for preliminary reports of these findings. This is a significant departure from a recent report suggesting eye-position modulations are too unreliable to be used for localizing stimuli (Xu et al., 2012). Once individual stimulus locations are extracted from eye position modulations, then in principle, by scanning the visual field with a series of saccades, the locations of multiple objects can be determined and placed into a spatial map.

Previous studies of eye position have generally not conducted a population analysis of the data. Rather, these studies have confined themselves to pointing out that receptive field properties of individual cells are consistent with the theoretical requirements for producing a coordinate transform, without actually using the population data to recover a spatial map. We are aware of no previous work that has attempted to do a general reconstruction of space based solely on eye position modulations of neural activity in any cortical area. Further, we believe this will be the first quantitative comparison of eye-position based visual space between dorsal and ventral visual streams.

## Materials and methods

### Behavioral task

**Figure 1:**
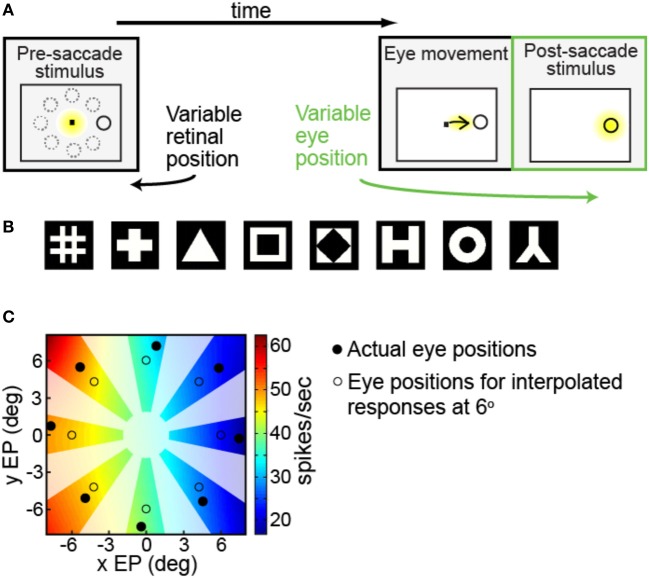
**Experimental and data analysis methods. (A)** Task design, showing sequence of events in a single trial. Yellow indicates where the monkey was fixating at each phase of the trial. After the monkey was stably fixating, the stimulus shape appeared randomly at one of eight peripheral locations (first panel). Dotted circles show possible target locations. The monkey immediately made a saccade to the stimulus (second panel). After the saccade (third panel, marked in green) the monkey was stably fixating the target (indicated by the yellow highlight) at some gaze angle. On different trials the target location randomly changed, so we could measure response to the same target stimulus for different eye positions. **(B)** Set of possible stimulus shapes. Preliminary testing of each cell indicated which of these eight shapes was the most effective stimulus for that cell, which was then used for subsequent measurements of eye-position modulations. **(C)** Example of interpolated/extrapolated responses used for multidimensional scaling (MDS) analysis, illustrated with data from one cell. Eight filled circles indicate locations where data was collected. Based on those data points an interpolated/extrapolated surface was fit (colored contours), providing an estimate of neural responses to a fixated stimulus over a continuous range of different eye positions. The colored contour map therefore forms a gain field for the cell, providing estimated responses at any arbitrary eye position. Eight open circles are an example set of interpolated/extrapolated eye positions used as input to MDS. Scale bar shows firing rates corresponding to the different colors in the estimated gain field. Only colored regions nearby data locations served as input locations for MDS calculations. That is, as no data came from blanked out regions (polar angles very different from data points, as well as the central area), interpolated values from those regions did not enter into MDS calculations.

We were interested in measuring the effects of different angles of gaze (i.e., eye position) on the responses of a neuron to the same fixated stimulus. Each trial began with the presentation of a fixation spot at the center of the visual display (Figure 1A, first panel). After the monkey was stably fixated on the fixation spot (yellow indicates where the animal was fixating), a stimulus of the preferred shape for the neuron appeared at one of eight peripheral locations (solid ring indicates target location; dashed rings indicate other possible locations). The animal was required to make an immediate saccade (indicated by the arrow) to the target in order to obtain a liquid reward (Figure 1A, second panel). When the eye position reached an invisible acceptance window centered around the target, the fixation point was extinguished and the target persisted on the screen for an additional 400 ms. Thus, after the saccade, the eye was stably fixated on the target at one of eight possible gaze angles (Figure 1A, third panel, yellow indicating animal's eye position). For this study we focus on neural responses during this last epoch of the trial (third panel), where across trials we can record the response of each neuron to the same fixated stimulus at different eye positions (gaze angles).

### Physiological preparation

Single-cell recordings were conducted on 2 male macaque monkeys (*Macaca mulatta*, 10 kg; *Macaca nemestrina*, 8 kg) while they performed the behavioral task described above. Prior to training a standard scleral search coil was implanted to monitor eye position. Recording was carried out in both LIP and AIT of each monkey from chambers implanted over the right cerebral hemisphere. The chambers for LIP, implanted first, were centered 3–5 mm posterior and 10–12 mm lateral. The chambers for AIT were implanted after recording from LIP had been completed and were centered 18 mm anterior and 18–21 mm lateral. Details of the surgical procedures have been described earlier (Sereno and Amador, 2006; Lehky and Sereno, 2007). All experimental protocols were approved by the University of Texas, Rutgers University, and Baylor College of Medicine Animal Welfare Committees and were in accordance with the National Institutes of Health Guidelines.

### Data collection and visual stimuli

The electrode was advanced while the monkey performed the task. All encountered cells that could be stably isolated were recorded from extracellularly and included in the raw data set. Either a platinum-iridium or tungsten microelectrode (1–2 MΩ, Microprobe) was used.

Stimuli were displayed on a 20-inch, 75-Hz CRT monitor with a resolution of 1152 × 864 pixels, located 65 cm in front of the monkey. The monitor subtended a visual angle of 27° × 36° (height × width). Other than the stimulus pattern or fixation spot, the screen was completely black. Beyond the monitor was a featureless 45 × 60 cm black screen (40° × 54°). With the exception of slight illumination from the screen patterns, the task was performed in darkness. The monkeys viewed the stimuli binocularly. The size of the fixation window around the fixation spot was 0.5° (half-width).

Prior to the start of data collection for each cell, preliminary testing determined the most effective shape stimulus for that cell (shape producing the highest firing rate) from amongst eight possible shapes (Figure 1B). After the most effective shape was found, further preliminary testing was used to find stimulus positions producing a robust response, using a grid of locations over a range of stimulus eccentricities and polar angles.

When testing eye-position modulations of neural responses, the target stimulus could appear at eight possible locations. The eight locations were arranged in a circle, all with the same eccentricity but different polar angles (Figure 1A, first panel). The chosen eccentricity, selected during the preliminary testing, reflected a balance between the two goals of maintaining a robust response and keeping similar stimulus locations for different cells. It did not necessarily maximize responses for each cell. The polar angles of the eight positions covered a full 360° in approximate 45° increments. Stimulus size for different cells ranged from 0.65° to 2.00° (mean: 0.8°), increasing with eccentricity.

After the monkey made a saccade to one of these eight target positions, the eyes were then at different gaze angles. We recorded each cell while the animal stably fixated the target at these different angles of gaze.

The goal was 12 correct trials per eye position, and median value of correct trials was 12, but in some cases we lost the cell before completing all desired trials. The number of trials was minimally 5, and almost always in the range 8–12. Only correct trials were further analyzed. Stimuli were presented in block mode. All eight positions were used once in random order to form a block, before being used again in the next block.

During data collection, we also tested and documented retinal spatial selectivity for the same cells by presenting the most effective stimulus shape at eight retinal positions while the monkey maintained central fixation. We have previously presented a comparison of retinotopic space in LIP and AIT based on these data (Sereno and Lehky, 2011).

### Data analysis

Data analysis methods here are essentially identical to what we previously used to study shape encoding and retinotopic space, but now applied to a different data set (eye position signals) and topic (eye-position based space).

Visual latency for each cortical area was determined from the pooled peristimulus histogram for all cells in a given cortical area, and defined as the time to half peak height of the histogram (92 ms for AIT and 70 ms for LIP). The time period used for the ANOVA and other analyses described below corresponded to a period when the eye was fixated on the target stimulus, immediately following the saccade from the central fixation spot. The period started 25 ms after the eye left the fixation window shifted by visual latency, and ended 200 ms after leaving the fixation window also shifted by visual latency. Thus, the analysis period began at 117 ms (AIT) or 95 ms (LIP) after the start of the saccade and its duration was 175 ms. Examination of eye coil traces showed that 25 ms was sufficient time for the eye to arrive and stabilize on the target stimulus. Overall, after shifting by visual latency the analysis window for AIT was 117–292 ms after the eye left the fixation window, and for LIP it was 95–270 ms. During the analysis window, the monkeys maintained stable fixation 92% of the time. We only included these stably fixated trials. Although not required to remain fixated on the target, on the majority of trials the animals maintained fixation until the target disappeared (400 ms after initial fixation).

Cells showing significant eye-position modulation of response (main effect of gaze angle) were identified using analysis of variance (ANOVA, *p* < 0.05). Subsequent analysis focused on those significant cells.

For each cell, a selectivity index for eye-position modulation was calculated:

(1) $$SI=\frac{r_{\max}-r_{\min}}{r_{\max}+r_{\min}}$$

with *r*_min_ and *r*_max_ being the minimum and maximum responses of the cell over all gaze angles tested. The values of selectivity index extended over the range 0.0–1.0, with higher values indicating greater eye-position modulation.

Because mean eccentricity of stimulus location in AIT and LIP was not identical, we performed an analysis of covariance (ANCOVA) to take eccentricity into account as a potential confounding factor when comparing the mean eye position selectivity for the two cortical areas. This essentially involved doing a separate linear regression for each cortical area for SI vs. eccentricity, and then determining if the two regression lines were significantly different or not.

The most important population level analysis performed was multidimensional scaling (MDS) (Shepard, 1980; Borg and Groenen, 2010). It was used to extract a global spatial map of stimulus locations based on a population of eye position responses. Our use of MDS for extracting spatial representations from neural populations has been extensively described in the context of retinotopic-based spatial signals (Sereno and Lehky, 2011); (see also Lehky and Sereno, 2011). The current application to eye-position based spatial signals is entirely analogous, and only a summary description will be given here. All MDS calculations were carried out with the Matlab Statistics Toolbox using the “cmdscale” command.

The MDS procedure was as follows. The responses of all cells in the data sample for a given brain area (LIP or AIT) to the stimulus at a particular eye position were combined to form a population response in the form of a neural *response vector* for that location. If there were *n* neurons in the population then the response vector had *n* elements in it. For each gaze angle, the same population of neurons had a different response vector. Thus, the starting point for the population level analysis is the high-dimensional population response vectors, with one response vector for each eye-position.

The next step was to calculate how much the response vector changed when eye position changed. This distance calculation was done for all possible pairs of response vectors (in other words, all possible pairs of eye positions), and the results were placed in a *distance matrix*. If there were *m* eye positions, then the distance matrix was an *m* × *m* square matrix. We used a correlation distance measure between response vectors, *d* = 1-*r*, where *r* was the standard Pearson correlation coefficient between pairs of response vectors. We used correlation distance rather than Euclidean distance because it was immune to non-specific shift in the overall firing rate of neurons.

MDS finds a low dimensional representation of the data in which the original distances between population response vectors are preserved as closely as possible. Given an *m* × *m* distance matrix whose elements are *δ*_*i,j*_ (distance between the neural response vectors for *i*th and *j*th eye positions), the MDS algorithm seeks to find *m* output vectors *x* such that ∑_*i* < *j*_(*d*(*x*_*i*_ − *x*_*j*_) − *δ*_*i,j*_)^2^ is minimized using an iterative optimization algorithm, where *d*() is the distance measure.

The output of MDS was a set of low-dimensional vectors, one for each eye-position. As gaze-angle is a two-dimensional variable, we were particularly interested in plotting the MDS output in two-dimensional space. If the relative positions of recovered points in this 2D space were isomorphic with the relative positions of the original gaze angles, then responses of the sample neural population successfully encoded eye position.

During the dimensionality reduction process, MDS produces a set of numbers called *eigenvalues*, which indicate how much of the variance of the data is captured by the low-dimensional representation. The number of eigenvalues is equal to the original dimensionality of the encoding neural population (number of neurons in our data set). To use these eigenvalues we first normalize them so that their sum equals one. The normalized eigenvalues directly give the fraction of variance that each dimension of the MDS output accounted for in the data. We sort the normalized eigenvalues in order of their magnitudes, from largest to smallest. If our small neural population were able to perfectly recover the original gaze angles in a 2D space that was isomorphic with the original gaze angles, all the variance in the data would be accounted for in the first two dimensions of the MDS output (first two sorted normalized eigenvalues) and all the other dimensions would have zero eigenvalues. If the first two dimensions are unable to capture all the variance in the data, the recovered low dimensional representation will be less isomorphic with the original gaze angles (less accurate) and we will find non-zero eigenvalues in the higher dimensions. In the figures, we only list the five largest eigenvalues.

A second way to quantify the accuracy of the eye-position representation recovered by the MDS analysis of population data is to use the *Procrustes transform* (Gower and Dijksterhuis, 2004; Borg and Groenen, 2010). The output of the MDS analysis is based purely on firing rates in a neural population, without any additional labeling of how the firing rate of each neuron relates to physical units of gaze angle. Therefore, the MDS output has firing-rate based values of eye position, whose numerical values are arbitrarily scaled relative to the values of physical eye positions given in degrees of visual angle. As neural representations of eye positions and physical eye positions were on different measurement scales we could not compare them directly. Rather we examined how relative values of recovered eye positions compared to the relative values of the actual eye positions.

The Procrustes transform is a method for determining the degree to which the relative values of two sets of points are isomorphic. To do that, the set of eight positions recovered from neural activity by MDS, **N**, based on inputs in units of spikes/s, was linearly transformed to numerically match as closely as possible the eight physical positions, **P**, specified in degrees of visual angle (i.e., to make the two sets of points, **N** and **P**, as congruent as possible, without changing the relation between points in **N**). The Procrustes transform minimizes the sum of squares for the errors T(**N**) − **P**, where T is a linear transform that includes translation, scaling, rotation, and reflection. In practice we used the “procrustes” command in the Matlab Statistics Toolbox.

To report how well the neurally-derived positions match the physical positions, we use an error measure called *stress*, which is a normalized sum of squared errors between T(**N**) and **P**:

(2) $$Stress=\sqrt{\frac{\sum_{i}\sum_{j}{\left(d_{ij}-{\hat{d}}_{ij}\right)}^{2}}{\sum_{i}\sum_{j}{\left(d_{ij}-\langle d_{ij}\rangle \right)}^{2}}}$$

In the equation, *d*_*ij*_ is the physical Euclidean distance between stimulus locations *i* and *j*, $\hat{d}$_*ij*_ is the distance recovered by MDS from the neural population representation, and 〈·〉 is the mean value operator. The Procrustes calculations were done in three dimensions, as in most cases three dimensions accounted for virtually all the variance in the MDS output. As the physical stimulus points were two-dimensional, we set the value of the third physical dimension equal to zero for all points when doing the calculations.

A small stress value signifies that the relative values of recovered and actual eye positions closely match and indicate an accurate neural representation of eye position. By convention, a stress value of less than 0.1 indicates that the MDS analysis has captured an accurate representation of the variable under consideration (analogous to the *p* < 0.05 convention in hypothesis testing statistics).

To produce the neural response vector at each eye position required by MDS, it is necessary for data from all cells in our data set to be recorded at the same eye positions. That is, when applying MDS to a population response vector, the method assumes that the entire population has received the same stimulus. In practice, as our cells were recorded one at a time, eye positions for different cells varied. We followed two mathematical procedures to deal with this issue.

The first approach illustrated in Figure 1C was to use the available data (indicated by the solid black dots) to create an interpolated gain field for each cell that estimated neural responses for nearby eye positions (indicated by the open circles) (cf., Zipser and Andersen, 1988, for previous use of interpolation to generate gain fields). Figure 1C shows an example interpolated gain field for a cell, in which a color code (see scale bar) indicates firing rates for different eye positions with the actual firing rate data collected for that cell indicated by the solid black dots. Using these interpolated gain fields we could then estimate neural response (i.e., open circles) at an identical eye position for all neurons in our sample, as required by MDS.

For each cell, we calculated a response *gain field* for all possible eye positions using bilinear interpolation (and extrapolation) from eye position data we had for that cell, which were responses to fixated stimuli at 8 positions. Bilinear interpolation is a generalization of linear interpolation to functions of two variables (in this case x and y coordinates of eye position). This procedure was strictly analogous to what we previously used for retinotopic space (Sereno and Lehky, 2011); (see also Zipser and Andersen, 1988). Using gain fields derived in this manner, we were able to estimate responses for each cell at any desired gaze angle within a limited range of eccentricities.

We performed two variant methods for creating interpolated gain fields. One variant used mean neural responses across all trials to create an interpolated gain field for each cell. The other used single-trial data as input, creating a separate interpolated gain field for each trial. Each single-trial response of a cell was treated as coming from a separate cell in the encoding population. Therefore, the total population in this case was found by summing over the number of cells in our sample multiplied by the number of trials for each cell.

The second approach to meeting the MDS requirement for identical eye positions for all cells in the population was to use averaging rather than interpolation. In the averaging method, for purposes of doing MDS calculations the actual eye position associated with each cell was replaced by a population average of eye position. When eye positions for a subset of cells fell within a small range of eccentricities (eye position locations across all cells within 3.3° of each other in AIT and 1.7° in LIP), we replaced the actual eye position eccentricity for each cell with the average eccentricity within that population. Based on these average eye positions we then proceeded with the MDS analysis. Here we shall emphasize the interpolation procedure as it offers more flexibility, as was discussed in Sereno and Lehky (2011), reserving the averaging procedure as a confirmatory method.

As MDS is a global method, all trials for all eye-positions for all cells included in the analysis contributed to each extracted point in the output (396 trials for AIT and 408 trials for LIP). The MDS procedure doesn't provide a means for calculating variances of the outputs directly using a formula. Therefore, to examine variability in results from the MDS analysis, we used bootstrap resampling, repeating the MDS analysis many times, each with a different resampling of the data. For each eye position in each cell, 100 bootstrap resamplings of individual trials were taken with replacement. These were used to generate 100 eye position gain fields for each cell using the interpolation method. The gain fields were in turn combined to form 100 populations, and an MDS analysis was performed on each population. Standard deviations of various MDS results could then be calculated from the set of resampled MDS analyses.

We were also interested if differences in results between AIT and LIP could be attributed to differences in the signal to noise ratio in their responses. To do this we calculated Fano factors for the data. The Fano factor is the ratio of variance to mean in the responses, *F* = *Var*(*x*)/*Mean*(*x*), where *x* is spike count during the spike train interval rather than spike rate. Variance was calculated from trial-to-trial variability in responses to the same stimulus. The Fano factor is actually a noise to signal ratio rather than a signal to noise ratio, but it serves equivalently for our purpose.

Finally, to compare the most responsive eye position in a cell's gain field with the most responsive retinal position for a stimulus, both over eight positions arranged in a circle, we calculated the circular correlation coefficient using the formula of Fisher and Lee (1983):

(3) $$r_{circ}=\frac{\sum \sin\text{​}\left(a_{i}-a_{j}\right)\sin\text{​}\left(b_{i}-b_{j}\right)}{{\left\{\sum \sin^{2}\left(a_{i}-a_{j}\right)\right\}}^{\frac{1}{2}}{\left\{\sum \sin^{2}\left(b_{i}-b_{j}\right)\right\}}^{\frac{1}{2}}}$$

where *a* and *b* are the two variables under consideration, and the summation is over 0 ≤ *i* < *j* ≤ *n* where *n* is the number of observations (eight in this case).

### Modeling

We created a model to demonstrate the principle that eye position and stimulus location can be recovered from a population of neurons with different gain fields, without a transform of spatial coordinates.

The shape of the gain field was a slanted sheet, similar to those described in monkeys (Andersen et al., 1985, 1990; Bremmer, 2000). Extreme gaze angles in a particular direction produced the highest firing rates, while extreme gaze angles in the 180° opposite direction produced the lowest firing rates. Central fixation produced an intermediate firing rate. The shape of the sheet was not planar, but had a sigmoidal curvature along one dimension. Using a sigmoidal profile was a convenient way of keeping neural responses in the model bounded to a limited range, in keeping with biophysical limitations.

Gain fields were described by the following equation, with relative firing rate *r* given by:

(4) $$\begin{array}{l} r=(\text{erf​}[\left(\sigma x\sin\text{​}\left(-\theta \right)-\sin^{2}\left(\theta \right)\delta \right)\\ \;+\left(\sigma y\cos\left(\theta \right)-\cos^{2}\left(\theta \right)\delta \right)]+1)/2 \end{array}$$

The neural activity is relative because it is on the arbitrary scale 0.0–1.0, rather than having realistic values. The terms inside the square brackets define a tilted 3D plane, with the third dimension being firing rate as a function of 2D eye position. A sigmoid cross section is imposed on that plane with the error function erf. Finally, the range of the erf sigmoid is shifted from the range [−1,1] to [0,1] by adding one and dividing by two, as firing rates cannot be negative. Figure 8 shows some example model gain fields produced by [Equation (4)], in which a color scale codes firing rate as a function of x and y eye position.

In [Equation (4)], the slope of the gain field, *σ*, defined how rapidly the field changed. The parameter *θ* defined the orientation or azimuth of the gain field tilt. The parameter *δ* controlled the location within the visual field of the inflection point of the sigmoid. The sigmoid inflection point is also the midpoint of the firing rate range in the gain field (0.5 in the range [0,1]). This last variable *δ* shifted the axis of anti-symmetry of the gain field away from passing through the central fixation point, with shift magnitude *δ* ranging from −1 to +1. The offset (shift) was measured as a fraction of the value of *σ*. Thus a given gain field was described by three parameters [*σ, θ, δ*]. The model did not include noise.

A population of model neurons with different gain fields was created by changing the values of [*σ, θ, δ*] for each neuron. We used *σ* = [0.250, 0.175, 0.122, 0.085, 0.059, 0.041, 0.029, 0.020], *θ* = [0, 45, 90, 135, 180, 225, 270, 315], and *δ* = [−1.00, −0.75, −0.50, −0.25, 0.00, 0.25, 0.50, 0.75, 1.00]. These values produced a total of 8 × 8 × 9 = 576 neurons in the population.

For stimulus conditions we chose a set of 32 eye positions, with eccentricity = [2°, 4°, 6°, 8°] and polar angles = [0°, 45°, 90°, 135°, 180°, 225°, 270°, 315°]. Each eye position produced a different pattern of activity in the population of 576 neurons. That is, each eye position produced a different response vector with length of 576. The 32 response vectors in the model were then analyzed using MDS in exactly the same manner described above for physiological data analysis, to produce a spatial map of relative eye positions.

Gain fields in the experimental literature are frequently fit by tilted planes (Andersen et al., 1985, 1990; Bremmer, 2000). A common parametric description of such linear gain fields is as a function of Euclidean coordinates in the visual field (*x, y*): *Ax* + *By* + *C*. We have parameterized the plane [terms inside square bracket of Equation (4)] differently here, in terms of variables that we believe are more intuitive for the issue at hand. Our parameterization of the plane is essentially in terms of spherical coordinates, in which the parameter *θ* is the azimuthal angle of spherical coordinates, and slope *σ* is a function of the elevation angle of spherical coordinates. Planar gain fields do not have anything equivalent to our third parameter, the shift parameter *δ*, as it is related to the sigmoidal non-linearity that warps the plane in our gain fields.

The best mathematical description of eye-position gain fields is still an unresolved issue. Although planar and quasi-planar gain fields have figured prominently in theoretical descriptions of data and approximate much published data (e.g., see Figure 1 in Bremmer, 2000; Figure 2 in Morris et al., 2013), actual gain fields can include more complex shapes. For example, Andersen et al. (1985) reported that 23% of gain fields did not contain a significant planar component.

## Results

We recorded from 80 cells in AIT and 73 cells in LIP. Histology confirmed that the LIP recording sites were indeed located on the lateral bank of the intraparietal sulcus. AIT cells came predominantly from areas TEav and TEad, with a few located in lateral perirhinal cortex (Brodmann area 36). The histology for these recordings has been described in detail previously (Lehky and Sereno, 2007). Mean latency of neural responses under our stimulus conditions was 92 ms in AIT and 70 ms in LIP.

Examining the data using analysis of variance (ANOVA), 41.3% (33/80) of AIT cells showed significant response selectivity for eye position. In LIP, 76.7% (56/73) of the cells were significantly eye-position selective. These percentages were determined at the *p* = 0.05 level of significance. From amongst cells that met the *p* = 0.05 significance criterion for spatial selectivity, LIP cells on average had greater significance (mean *p* = 0.0046) than AIT cells (mean *p* = 0.0142).

For all recorded cells, mean stimulus eccentricity in AIT was 4.3° (range: 2.1°–10.0°), and in LIP it was 10.9° (range: 6.3°–17.7°). For the subset of cells with significant eye-position selectivity, mean stimulus eccentricity in AIT was 3.9° (range: 2.1°–6.9°). For LIP cells with significant eye-position selectivity, mean stimulus eccentricity was 11.2° (range: 6.3°–17.7°).

Different cells had different mean responses over all eye positions tested. For AIT cells with significant eye-position modulation, average response ranged from 3.4 to 96.0 spikes/s, with a grand average over all cells of 21.2 spikes/s. For LIP cells with significant eye position modulation effects, the range was 0.4–84.6 spikes/s, with a grand average of 19.7 spikes/s. The difference in grand average responses for AIT and LIP was not significant under a *t*-test (*p* > 0.7) and a Wilcoxon rank sum test (*p* > 0.35). While there was no significant mean firing rate difference between LIP and AIT for stimuli fixated at different gaze angles, we have previously found that LIP had a significantly higher mean firing rate than AIT for stimuli presented at different retinal locations with gaze angle centrally fixed (Sereno and Lehky, 2011).

**Figure 2:**
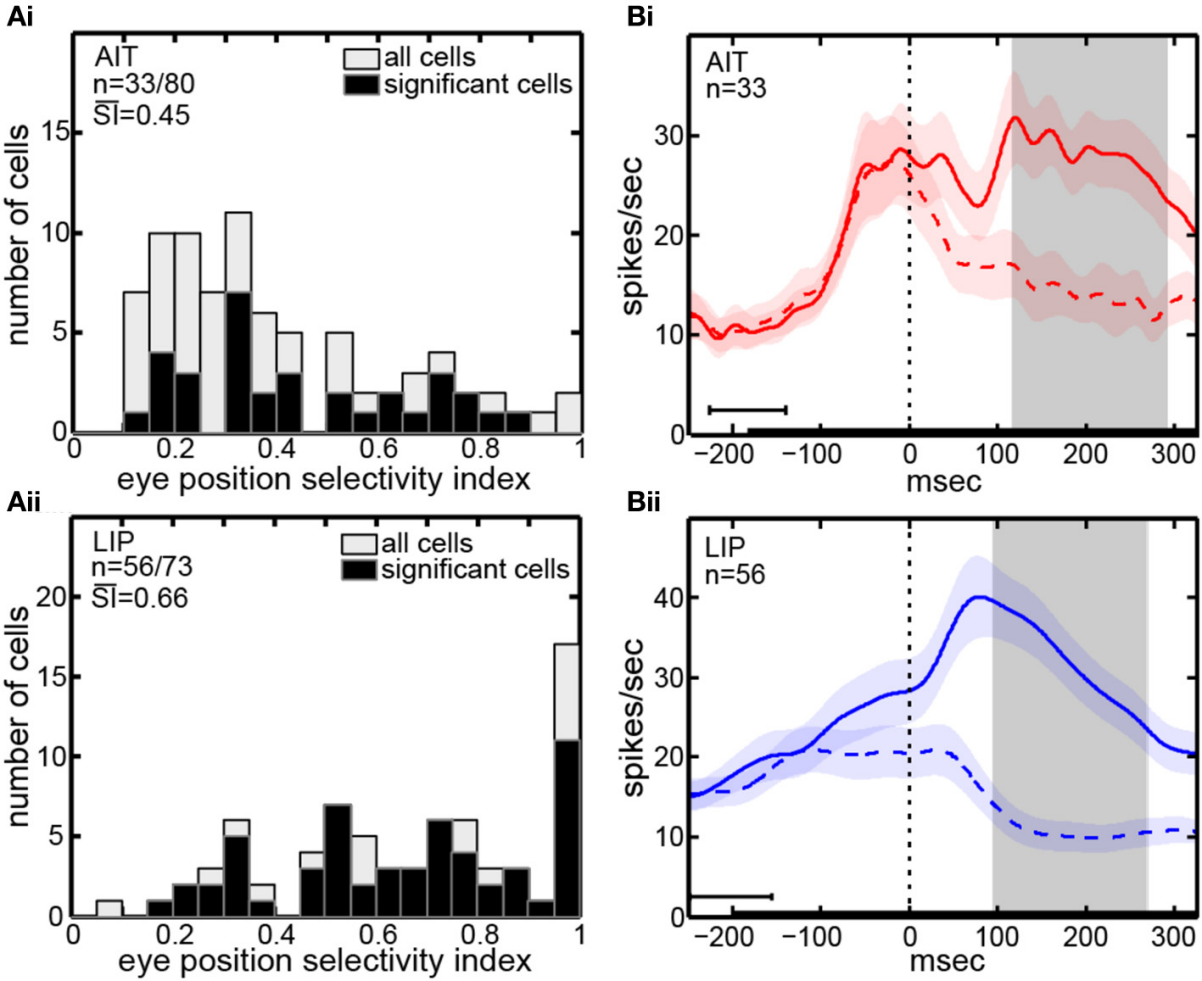
**Eye position selectivity. (A)** Spatial selectivity index (SI) histograms for AIT (upper panel, **Ai**) and LIP (lower panel, **Aii**). The SI was calculated for each recorded cell using [Equation (1)]. Included are *SI*-values for all recorded cells (open bars; second *n*-value) as well as for only those cells showing significant spatial selectivity (black bars; first *n*-value). Mean *SI*-value for spatially significant cells is also shown in each panel indicating the average magnitude of the effects. **(B)** Average time course of responses (PSTH), aligned to start of saccade to target for AIT (upper panel, **Bi**, red lines) and LIP (lower panel, **Bii**, blue lines). Time course calculated over all cells showing significant selectivity for eye position, at the most responsive eye position (solid line) and least responsive eye position (dashed line). Shaded regions around lines show standard errors of responses over cells in the sample population. Zero time marks when the eye left the central fixation window during saccade to target. Black bar at bottom shows target presentation period, with error bar indicating the standard deviation of target onset before saccade to target. Gray shaded region shows the time period used for data analyses (ANOVA, SI, and MDS), beginning 25 ms after start of saccade and ending 200 ms after start of saccade, both times are shifted by the average latency of the visual responses in the respective cortical area.

The eye-position selectivity index (SI) [Equation (1)] was calculated for each cell in our sample. Figure 2A presents histograms of the *SI*-values. The mean SI for AIT cells with significant eye-position selectivity was 0.45. LIP cells with significant eye position selectivity had a higher mean *SI*-value of 0.66. In other words, LIP cells showed greater modulation of their responses as gaze angle changed than AIT cells. The difference between mean SI in AIT and LIP was significant at the *p* = 0.001 level under a Wilcoxon rank sum test.

We used analysis of covariance (ANCOVA) to examine the influence of two factors on the values of the eye-position SI in our sample of neurons: (1) eccentricity, and (2) brain area (AIT or LIP). The ANCOVA results showed that eccentricity was a significant factor affecting SI (*p* = 0.026), and that brain area was not a significant factor (*p* = 0.353). In other words, there was no significant difference in the magnitude of gaze angle modulation in AIT and LIP for our sample, once different eccentricities of cell samples in the two areas were taken into account.

The time course of visual responses in AIT and LIP (PSTHs) are plotted in Figures 2Bi,ii, respectively, averaged over all cells showing significant selectivity for eye position under ANOVA. Separate plots indicate average response at the best eye position (solid line) and worst eye position (dashed line) in each brain area.

A notable difference between the two brain areas is that LIP cells at their least responsive eye position are on average suppressed relative to baseline, whereas AIT cells are not. We have previously reported a similar suppression in LIP cells at their least responsive retinotopic position (Sereno and Lehky, 2011). In particular, similar to what we noted with respect to retinotopic responses, the presence of suppressed responses in LIP serves to increase the dynamic range of responses to different angles of gaze (target spatial positions) and may contribute to the higher eye-position SI observed in LIP (Figure 2A). Interestingly, although suppressed responses in population averages exist in LIP but not AIT for both retinal and eye position spatial signals, the opposite occurs in the shape domain. That is, the least effective shape leads to suppression relative to baseline in AIT, but not LIP (Lehky and Sereno, 2007).

### Comparing retinotopic and eye-position spatial modulations

In our task, the eye changed position by moving to a target at a particular retinotopic location. We were interested to know whether the eye position producing the strongest response corresponded to the retinotopic target location producing the strongest response to the same stimulus. In other words, was there any relation between the spatial topography of a cell's eye-position gain field and the topography of its retinotopic responses.

We found that the most responsive eye position matched the most responsive retinal target position 17.5% of the time in AIT and 19.2% of the time in LIP. Chance level was 12.5% for the eight locations used. Bootstrap resampling showed that the observed correspondence did not significantly differ from chance in either AIT (*p* = 0.15) or LIP (*p* = 0.09). As retinal target positions (and therefore subsequent eye positions) were arranged in a circle at eight polar angles, we also calculated the circular correlation coefficient given by [Equation (3)] (Fisher and Lee, 1983) between retinal responses and eye position responses. In AIT the circular correlation coefficient had a mean −0.0097 and standard deviation 0.19 across all cells, while in LIP the mean was −0.0030 and standard deviation 0.21, all calculated using Fisher weighted correlation coefficients.

Thus it appears that the spatial organization of responses across different retinal locations is very different from the spatial organization of responses for different eye positions. The fact that they are so different suggests that our post-saccadic gain field measurements are not contaminated to a significant extent by residual pre-saccadic receptive field properties, which might have remained because of sluggish neural dynamics in updating responses after changing eye position.

We also compared the spatial selectivity indices for retinotopic position and eye position across all cells. The correlations were moderately high, being 0.59 for AIT and 0.67 for LIP. This indicates that cells that were more highly modulated by changes in stimulus retinal position also tended to be more highly modulated by changes in eye position, even though the spatial arrangement of modulations was different in each case.

### Multidimensional scaling

**Figure 3:**
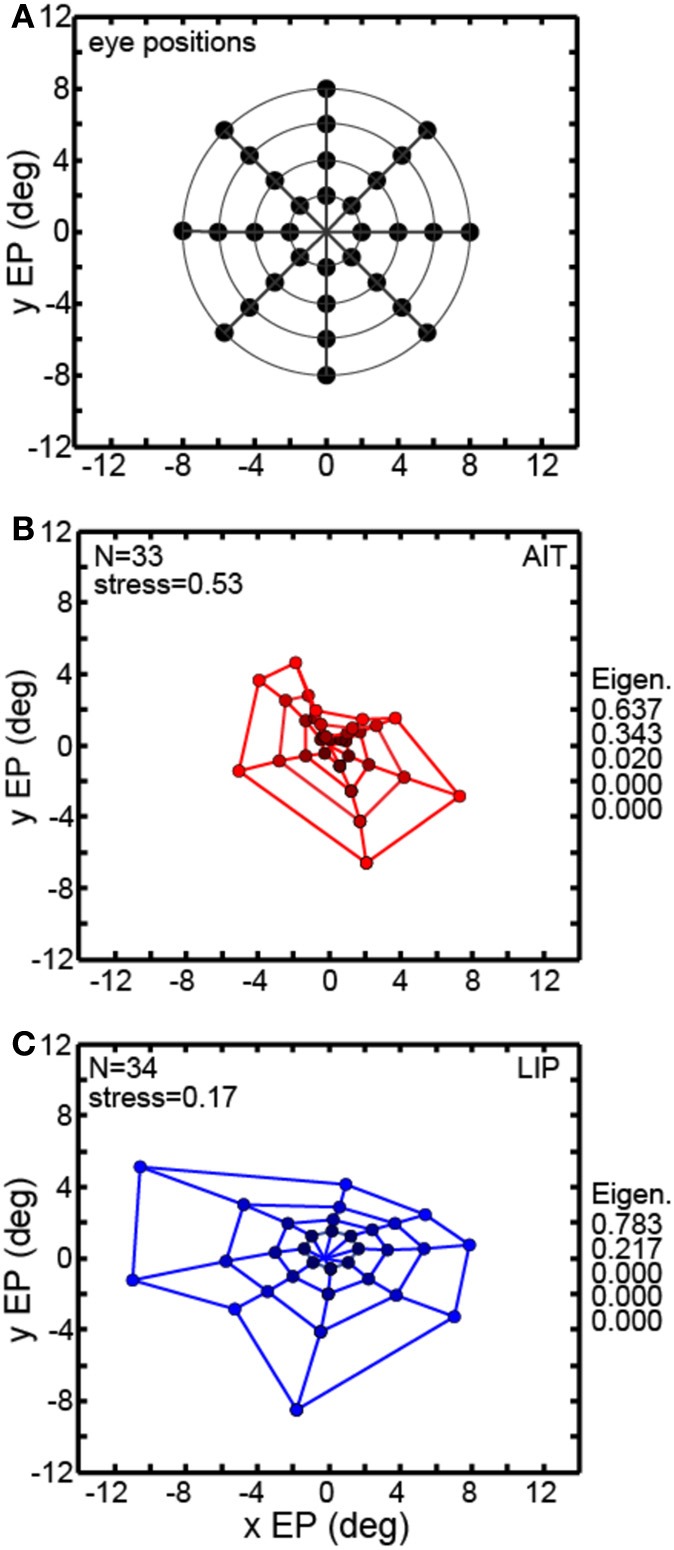
**Recovery of eye positions from neural population activity, using a global stimulus configuration and multidimensional scaling (MDS) analysis**. MDS analysis was based on using interpolated neural responses from recorded neurons that had significant spatial selectivity under ANOVA. This analysis used mean neural response across trials. **(A)** Set of eye positions used as input configuration for MDS analysis. It consisted of 32 points arranged in a polar grid. The center of the grid corresponded to central fixation. As illustrated, the eye positions were arranged over four eccentricities with visual angles of [2°, 4°, 6°, 8°]. At each eccentricity, eight locations were arranged in an iso-centric circle at 45° polar angle increments. Each of the 32 eye positions produced a different activation pattern (response vector) in the population of neurons in our data set. Lines connecting the positions merely help illustrate iso-eccentricity positions and iso-polar angles as well as highlight the overall symmetry of the spatial configuration. **(B)** Configuration of eye positions recovered from AIT data, shown in red. **(C)** Configuration of eye positions recovered from LIP data, shown in blue. There is less distortion apparent in the spatial layout of the LIP grid compared to AIT and the LIP stress value is lower than in AIT, indicating a more accurate global recovery of eye positions. For both panels **(A)** and **(B)**, color darkens with decreasing eccentricity, to aid visualization. Also for both panels, normalized MDS eigenvalues are displayed.

Multidimensional scaling forms the centerpiece of the data analyses here. To deal with the mathematical requirement of MDS that eye positions for all cells be identical, spatial interpolation from the available data points was performed before the MDS analysis. An example interpolated gain field is shown in Figure 1C, with neural responses for different eye positions indicated by a color scale. Responses at identical eye positions for all cells in our sample, derived from interpolated gain fields, were then used as input to MDS. Only cells having significant spatial selectivity under ANOVA and which had an eye-position eccentricity of less than 10° were included, producing population size *n* = 33 for AIT and *n* = 34 for LIP. Responses at 32 eye positions (four eccentricities and eight polar angles; positions arranged in a polar grid as illustrated in Figure 3A) were calculated for each cell using its interpolated gain field. Lines connecting these positions have no significance other than to aid visualization, helping to illustrate iso-eccentricity positions and iso-polar angles as well as highlight the overall symmetry of the spatial configuration. These 32 responses for each cell in the AIT and LIP populations were used as input to MDS.

The grid of eye positions recovered by MDS analysis of AIT and LIP neural populations is shown in Figures 3B,C (AIT-red; LIP-blue; with dot color darkening with decreasing eccentricity). Again, lines connecting these recovered eye positions merely help illustrate the layout and distortions in the recovered spatial configurations. In both brain areas the information contained within their respective neural populations was sufficient to extract an organized spatial map of eye positions. As the stimulus was always at fixation for all eye positions, recovering eye position was equivalent to recovering stimulus target location. Procrustes analyses comparing relative eye positions in physical space with relative eye positions encoded by neural populations showed that the spatial map recovered in LIP was more accurate (less distorted) than the AIT spatial map. That was indicated by the lower stress value for the LIP analysis (stress = 0.17 ± 0.02) compared to AIT (stress = 0.53 ± 0.08). Stress standard deviations are based on MDS analysis performed on bootstrap resampling of the data. Stresses in AIT and LIP were significantly different at *p* = 0.0022, also based on bootstrap resampling.

The normalized eigenvalues in Figures 3B,C give the fraction of variance in the data accounted for by each of the mathematical dimensions produced by the MDS analysis. The first two mathematical dimensions are interpreted as corresponding to the two physical dimensions of gaze angle. To be perfectly isomorphic with physical space, the first two normalized eigenvalues should both be equal to 0.5, and all the other dimensions equal to zero. However, for both AIT and LIP there is a large inequality in the two eigenvalues, corresponding to a slight vertical squashing of the eye position map apparent in the plots. The third dimension in AIT is non-zero, indicative of a level of distortion in the AIT representation of eye position not present in the LIP representation.

Neurons in LIP were able to extract eye positions more accurately than those in AIT, as shown by lower stress values (Figure 3). To investigate if that was because the relative amounts of noise and signal were different in LIP and AIT, we calculated Fano factors for the data. The Fano factor is the ratio between variance and mean of spike counts in the data, with variance calculated here across trials. A separate Fano factor was calculated for each of the eight eye positions for each cell, and the set of all Fano factors for a given brain area (AIT or LIP) were placed in one pool.

The distributions of Fano factors for spike count in both our AIT and LIP samples were highly skewed to the right, but a log transform normalized those distributions. For AIT, the geometric mean of the Fano factors was 0.76 and the median was also 0.76. For LIP the geometric mean was 0.73 and the median was 0.76. The difference in Fano factors for AIT and LIP was not significant, with *p* = 0.48 for a *t*-test and *p* = 0.51 in a Wilcoxon rank sum test, using log transformed Fano factors. We conclude that the difference between the MDS results for AIT and LIP are not due to differences in the relative noisiness in the two areas.

**Figure 4:**
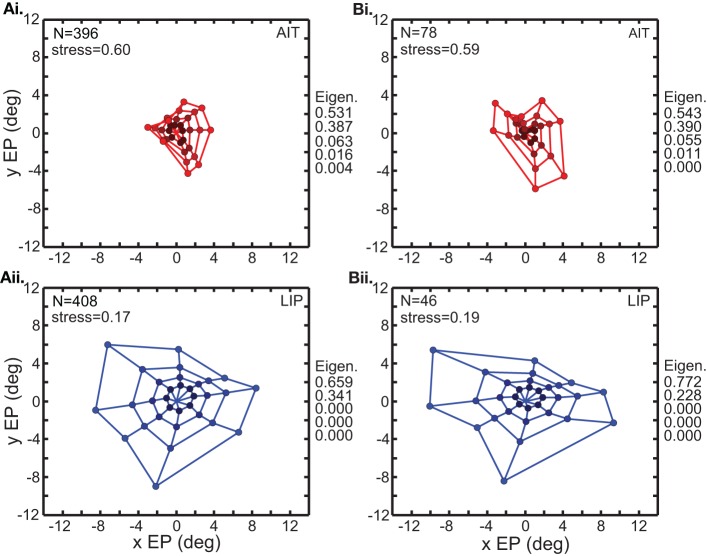
**Two variations of the multidimensional scaling analyses in Figure 3**. Conventions are the same as in Figure 3, with red points indicating results from AIT and blue points results for LIP. **(A)** Recovery of eye positions from neural population activity, using single-trial results rather than mean results across trials. Each single-trial response for a given cell was treated in the MDS analysis as a separate cell in the population. **(B)** Recovery of eye positions from neural population activity, using all cells in the data set rather than only cells that had significant eye-position modulation. Normalized MDS eigenvalues indicated to the right of each panel.

Figure 3 comprises the basic MDS results, and what follows in the rest of the MDS section is a series of controls and elaborations on those results. Two variants of the analysis in Figure 3 are shown in Figure 4. In the first (Figure 4A), input to MDS is single-trial responses rather than mean response over all trials as in Figure 3. In the second (Figure 4B), the input to MDS is the responses from all cells, and not just cells having statistically significant eye-position modulations as in Figure 3.

Figure 4A shows that a spatial map of eye positions (target locations) can still be recovered from single-trial data. The signal-to-noise ratio in single trials appears sufficient to perform population coding of location. For purposes of doing MDS, each single-trial was treated as the response of a different cell, so the single-trial MDS was based on a noisier but much larger (several hundred cells) effective population than the mean-response MDS. Because the single-trial MDS incorporated information from all trials, it was still pooling trials, but pooling the information in a different way than the mean-response MDS analyses. It is likely that real neural populations are vastly larger than our sample. By treating multiple trials of a single cell as single trials of multiple cells, what Figure 4A shows is that a large population (several hundred cells) of noisy cells can still effectively decode eye position.

Figure 4B shows the results of MDS analysis when all cells in our sample were used and not just significant cells. Comparison between Figure 3 and Figure 4B indicates that the inclusion of non-significant cells had only a minor effect.

**Figure 5:**
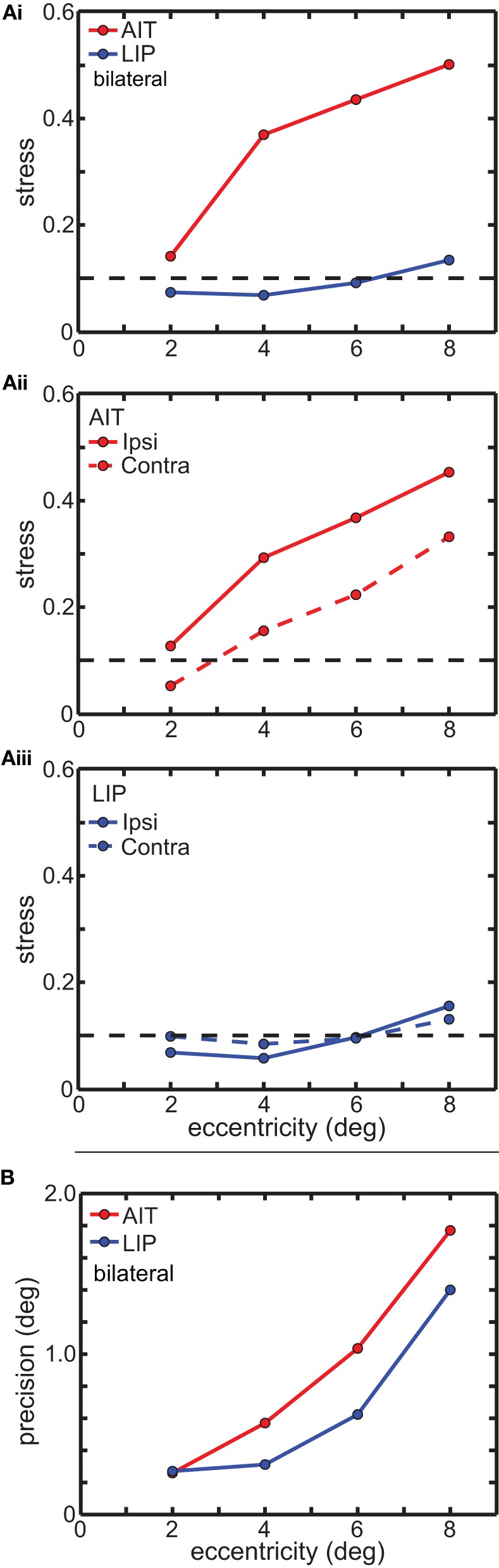
**Global and local error measures for multidimensional scaling analysis (MDS) results in Figure 3. (A)** Global error (stress) as a function of stimulus eccentricity. Stress values below 0.1 (dashed line in each panel) indicate highly accurate spatial representations. **(Ai)** Comparison of stress in AIT and LIP using bilateral (ipsilateral and contralateral) data. **(Aii)** Stress for AIT representations for ipsilateral and contralateral eye positions. **(Aiii)** Stress for LIP representations for ipsilateral and contralateral eye positions. **(B)** Local error (precision) as a function of stimulus eccentricity for both AIT (red points) and LIP (blue points). Precision is the standard deviation of recovered eye position, as determined by bootstrap resampling of the data. Precision was individually calculated for each eye position in Figures 3B (AIT) and **3C** (LIP), and then for each area averaged over all eye positions having the same eccentricity.

In Figure 5 we examine error in the MDS results as a function of eye position eccentricity. Two error measures are presented. The first (Figure 5A) is a global error measure for the MDS output as a whole, namely *stress*, which was described in the Materials and Methods section. The second (Figure 5B) is a local error measure for individual points in the MDS output, which is simply the standard deviations of those points, which we call *precision*.

Figure 5A shows the effect of eye-position eccentricity on the global accuracy of the recovered spatial maps in AIT and LIP. For this analysis, instead of performing MDS using a grid of eye positions as in Figure 3 we did MDS separately for each of the four rings in that grid, located at four different eccentricities. Stress is a global measure of accuracy because it depends on relative positions of the eight points in each ring, not the individual points in isolation. Low stress means that the eye positions recovered by MDS closely matched the shape of a circle, while high stress means that the MDS output formed a very distorted circle.

The results are shown in Figure 5Ai, plotting stress vs. eccentricity. This demonstrates that eye-position maps become less accurate (greater stress) as eccentricity increases for AIT, but not LIP. At any given eccentricity the LIP map was more accurate than the AIT map. The difference in stress between LIP and AIT was significant at all eccentricities except the smallest, 2°, based on bootstrap resampling of neurons in the data set. Going from 2° to 8° in Figure 5Ai, *p*-values for the difference were 0.24, 0.0049, 0.0013, and 0.0003.

We also compared global accuracy of the eye-position spatial maps when eye position was ipsilateral or contralateral to the recorded hemisphere. In AIT, the contralateral map was more accurate than the ipsilateral one (Figure 5Aii). In LIP there was little difference between ipsilateral and contralateral results (Figure 5Aiii), with stress remaining independent of eccentricity for both.

Local error measures, the precisions (standard deviations) of recovered eye positions in Figure 3, are shown in Figure 5B. These were calculated from bootstrap resampling of the data, and as these are local measures the calculations were done individually for each eye position. Because precision was similar for all eight eye positions at each eccentricity, we plotted the average precision at each eccentricity (indicated by the points in Figure 5B; red points = AIT; blue points = LIP) as a function of eccentricity. As with stress (Figure 5A), precision increases with eccentricity, showing that the quality of eye position spatial representations get worse for larger gaze angles in both LIP (blue line) and AIT (red line).

**Figure 6:**
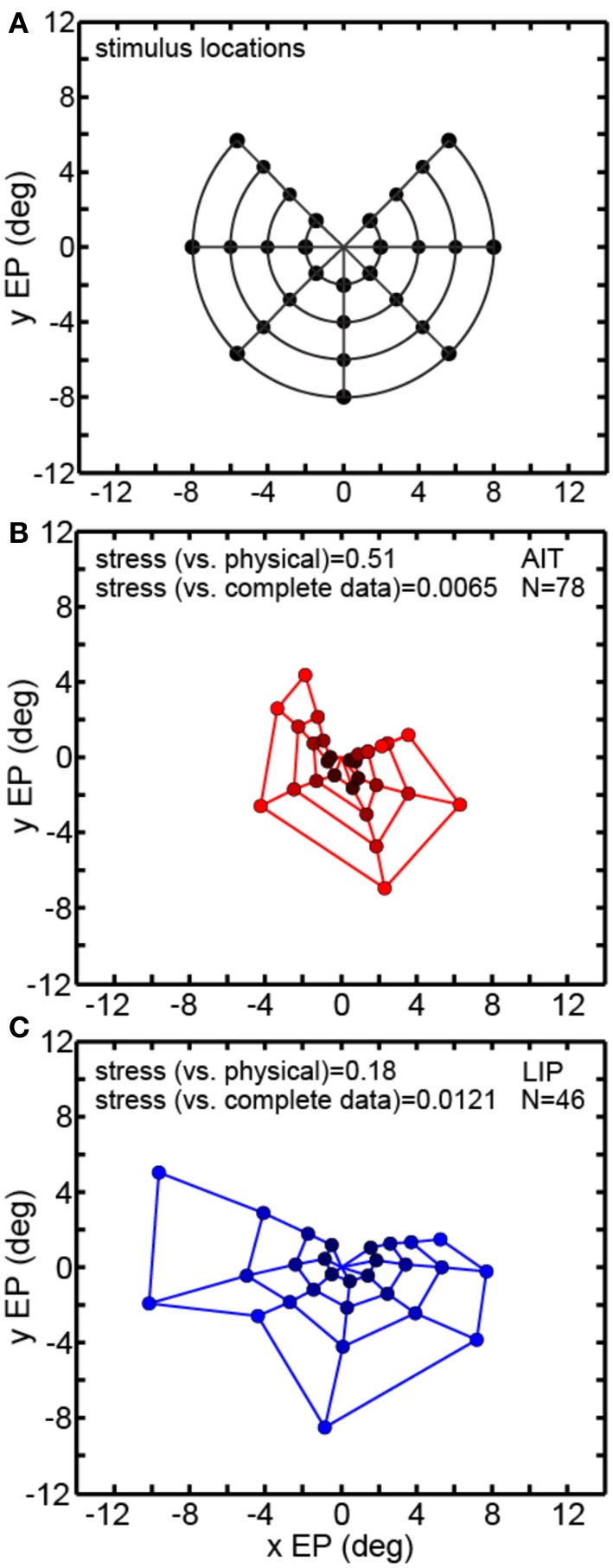
**Multidimensional scaling recovery of eye positions using a partial set of inputs**. Conventions are the same as in Figure 3. **(A)** Eye positions used as input configuration for MDS analysis, with one wedge or sector removed compared to the complete set shown in Figure 3A. **(B)** Configuration of eye positions (red points) recovered from AIT using partial data set. **(C)** Configuration of eye positions (blue points) recovered from LIP using partial data set. Colors darken at lower eccentricities to aid visualization. Both data panels give stress between recovered eye positions and physical eye positions, as well as stress between recovered eye positions for full and partial data sets. Small stress values between full and partial data indicate that eye position recovery is not highly sensitive to the precise composition of the global configuration used as input to MDS.

Figure 6 examines the robustness of MDS decoding by removing data (inputs) from some points in the eye position grid and seeing how that affects decoding of the remainder of the eye positions. As MDS is a global rather than local decoding method, removing some of the data could have deleterious effects that propagate to the decoding of other eye positions. By removing some of the eye positions we also break the circular symmetry of the inputs, which might also possibly produce some sort of special-case configuration advantage. Specifically, we removed one of the eight polar angle locations in the eye position set and checked how that perturbed the recovery of the other seven polar angles. Removing one polar angle produced a bull's-eye polar grid pattern with a wedge missing (Figure 6A).

Removing one polar angle from the stimulus set had a minimal effect on decoding the points at the seven remaining polar angles (Figures 6B,C). Directly comparing eye positions extracted by MDS for corresponding points in the full (Figure 3) and one-angle missing sets (Figure 6) with each other, we found stress values to be 0.0065 for AIT and 0.0121 for LIP (averaged over all eight comparisons—i.e., a pattern with one of the eight wedges missing vs. the full pattern for each comparison). This is an indication that MDS results are robust to changes in the selection of the input set.

**Figure 7:**
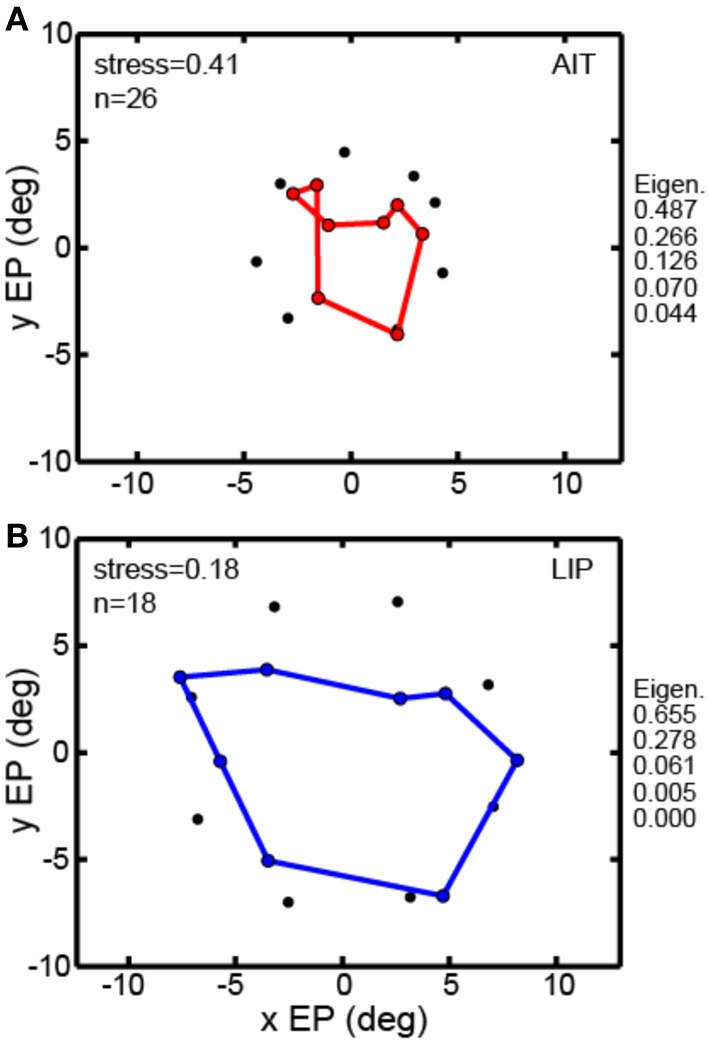
**Multidimensional scaling recovery of eye positions from population data using the averaging method with a subset of cells rather than interpolation method employed in Figure 3. (A)** Configuration of eye positions recovered from AIT (red points). **(B)** Configuration of eye positions recovered from LIP (blue points). This averaging method replicates the observation found using the interpolation method; namely, that LIP neurons produce a more accurate representation of eye position than AIT (lower stress in LIP than AIT). Normalized MDS eigenvalues indicated to the right of each panel.

All the MDS analyses described above were done using interpolated gain fields. In Figure 7 we examine a second way of dealing with the MDS mathematical requirement that eye positions for all cells be identical, using averaging rather than interpolation. For the averaging method, eye positions within a narrow band of eccentricities were all treated as if they had the same eccentricity given by the population average. For AIT, all cells in the eccentricity range 3.6°–6.9° were treated as located at eccentricity 4.4° (*N* = 26). For LIP, all cells in the eccentricity range 6.3°–8.0° were treated as located at eccentricity 7.5° (*N* = 18). For this method, rather than having a grid of eye positions as in Figure 3, there was a single ring of eight eye positions at the indicated eccentricity.

The averaging method (Figure 7) was able to extract a spatial map of eye positions with stress (spatial distortion) values comparable to those found by the interpolation method. For AIT stress was 0.41 (Figure 7A) and for LIP it was 0.18 (Figure 7B). Similar to the findings using the interpolation method, stresses in AIT and LIP under the averaging method were significantly different (*p* = 0.03), based on MDS analysis performed on bootstrap resampling of the sample of cells in the data.

Thus, the observation that eye position spatial maps could be extracted from visual responses in cortical populations was replicated using two approaches to performing the MDS calculations, interpolation, and averaging. The observation that LIP produced more accurate eye position maps than AIT was also replicated under these two approaches. The fact that the basic observations are reproducible under two independent analysis methods strengthens the case that these findings are not an artifact of using a particular approach.

### Modeling

We constructed a model to recover eye positions from a population of model neurons with diverse gain fields, analogous to what was done with the data from LIP and AIT neurons. Such a model lays the foundation for studying the effects of different gain field characteristics on the representation of eye-position space under well defined parametric conditions, which are more difficult, if not impossible in the laboratory.

**Figure 8:**
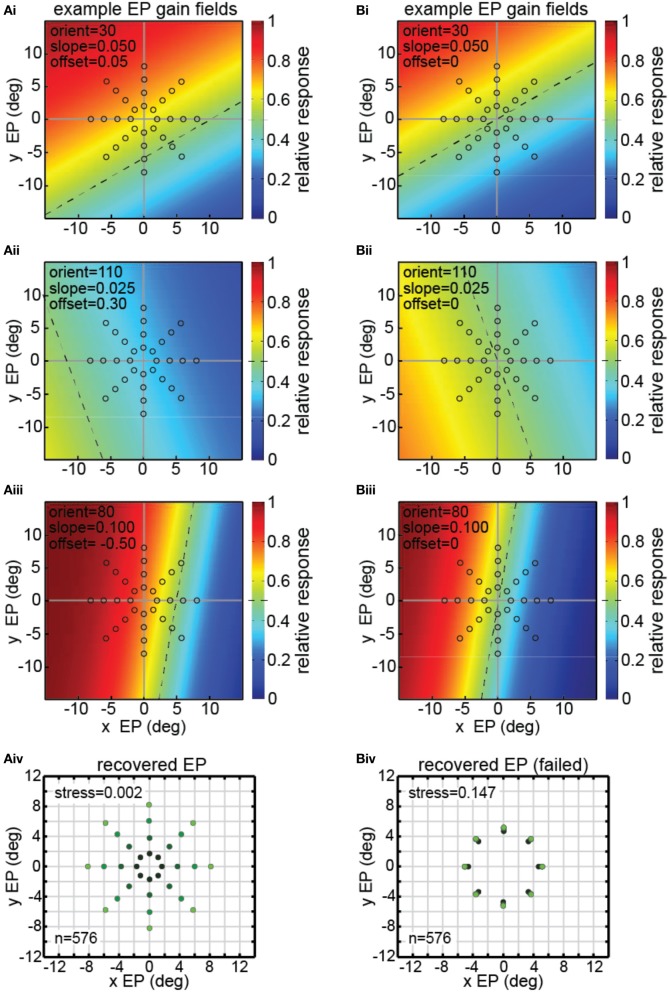
**Model results showing decoding of eye position, using multidimensional scaling on a population of model neurons with diverse eye-position gain fields**. Population consisted of 576 neurons, each with a different gain field. Gain fields were defined by three parameters: slope, orientation, and offset, defined in [Equation (4)]. The color (see scale at right) represents the relative response rate at each eye position. The midpoint firing rate (green) within each gain field (0.5 within the range 0.0–1.0) is shown by a dashed line. **(A)** Successful decoding of eye position by the model including asymmetric gain fields with large non-zero values of the offset parameter *δ* = [−1.00, −0.75, −0.50, −0.25, 0.00, 0.25, 0.50, 0.75, 1.00]. **(Ai–iii)** Three examples of model gain fields with large offsets. Dashed lines do not pass through the origin (central fixation), indicating model neurons included asymmetric gain fields. Open circles indicate eye position locations used as input to MDS analysis, consisting of 32 different eye positions. **(Aiv)** Multidimensional scaling model results for recovering eye positions, including gain fields with asymmetric gain fields produced using large offsets. Recovered eye positions closely correspond to physical eye positions depicted by open circles in panels **(Ai–Aiii)**. Low stress value indicates the model was able to recover a very accurate representation of relative eye positions. To aid visualization of the spatial configuration of recovered eye positions, the color in the recovered eye position points darkens with decreasing eye position eccentricity. **(B)** Unsuccessful decoding of eye position by the model using anti-symmetric gain fields produced by small (near zero) offsets (offset parameter, *δ* = [−0.100, −0.075, −0.050, −0.025, 0.000, 0.025, 0.050, 0.075, 0.100]. **(Bi–Biii)** Three examples of model gain fields with zero offsets. Dashed lines pass through the origin (central fixation), indicating anti-symmetric gain fields. Open circles indicate spatial locations used as input to MDS analysis, consisting of a stimulus fixated at 32 eye positions. **(Biv)** MDS modeling results using nearly anti-symmetric gain fields produced using small (near zero) offsets. In this case the model failed to extract accurate eye positions, creating a high stress value, as recovered positions did not closely correspond to physical eye positions depicted by open circles in panels **(Bi–Biii)** As in **(Aiv)**, color in the recovered eye position points darkens with decreasing eye position eccentricity. In this case, however, the different recovered eye position eccentricities lay nearly on top of each other.

There were 576 neurons in the model population, each with a different gain field described by [Equation (4)]. Example gain fields are shown in Figure 8, with neural response as a function of the x and y coordinates of eye position coded in color (see scale bar at right side). Each gain field is described by different values for the parameters *slope, orientation* (azimuth), and *offset* (shift), which correspond to the variables [*σ, θ, δ*] in [Equation (4)]. The dashed lines in the gain fields indicate the inflection point of their sigmoidal shape. That is, the dashed lines represent the center axis where the model gain field has its midpoint response value of 0.5 (denoted by green in the color scale) over the relative response range 0.0–1.0. The value of the offset parameter sets the position of the dashed line relative to central fixation at [0,0]. Zero offset places the dashed line passing through [0,0], producing anti-symmetric gain fields (gain fields centered around central fixation). Non-zero offsets place the dashed line away from the central fixation point, producing asymmetric gain fields with respect to central fixation. The three gain fields in Figure 8A have non-zero offsets and therefore are asymmetric. The three gain fields in Figure 8B have zero offsets and are anti-symmetric and centered around central fixation.

The stimulus and eye position conditions underlying the model were the same as for the experimental data: neural response to an identical visual stimulus fixated at a variety of eye positions (gaze angles). The configuration of eye positions used as input to the MDS analysis is shown by the open circles in Figures 8Ai–iii,Bi–iii. Each of those 32 eye positions produced a different response vector in the model neural population, which could then be decoded by the MDS procedure.

The three example gain fields in Figure 8A had asymmetric gain fields with large offset values *δ* = [−1.00, −0.75, −0.50, −0.25, 0.00, 0.25, 0.50, 0.75, 1.00]. The synthetic data produced by the model with such gain fields were analyzed in an identical manner to that used with the actual data (MDS followed by a Procrustes analysis). As shown in Figure 8Aiv, we were able to recover a highly accurate spatial map of eye positions from those synthetic data (stress = 0.002).

However, not all gain field population characteristics were successful in recovering eye positions (or equivalently, recovering stimulus locations), importantly indicating that this simple model is not tautological. If the gain fields were very close to anti-symmetric (small offset values *δ* = [−0.100, −0.075, −0.050, −0.025, 0.000, 0.025, 0.050, 0.075, 0.100]), as in Figure 8B, recovery of eye positions was quite bad (Figure 8Biv), much worse than for the asymmetric gain fields in Figure 8Aiv. Note that there is little correspondence between the recovered eye positions in Figure 8Biv and the physical eye positions shown by the open circles in Figures 8Bi–iii.

We do not know whether the asymmetric/anti-symmetric distinction made here is of fundamental importance for real-world gain fields. Interestingly, the two types of gain fields happened to have strikingly different capabilities for encoding eye position, under the simple mathematical description of gain fields we chose for the model. Basically, the modeling demonstrated that some gain field characteristics were better than others for decoding eye position signals. Possibly other gain field characteristics besides anti-symmetry may be important in decoding eye position in actual brains. Hence, the modeling may point to what gain field properties to measure in real cells. Further development and investigation of different parameters of the model are sure to provide insight into our understanding of eye position signals, as well as the representation of space in different brain areas more generally.

## Discussion

We demonstrate here for the first time that spatial maps of eye position can be extracted directly from visual cortical responses in both a ventral structure (AIT) as well as a dorsal structure (LIP) (see also Sereno, 2011; Sereno and Lehky, 2012 for preliminary reports). Extracting eye position is equivalent to extracting the location of a fixated stimulus. Our success in population decoding of eye position signals stands in contrast to recent reports based on recordings in the dorsal stream that such modulations were unreliable (Xu et al., 2012) or inaccurate (Morris et al., 2012) when used for calculating target position during the period following an eye movement. Secondly, in a first direct comparison of regions of the brain where we expect dorsal/ventral differences in visual processing to be highly salient, we demonstrate significant differences in the representation of eye-position based space. We further elaborate on the meaning and consequences of these main findings as well as others below.

### Method: advantages of population coding vs. single cell analyses

In this study, we used a population decoding technique, MDS, to extract gaze angle and stimulus location from a population of cells modulated by eye position. We believe a population approach to eye-position signals, going beyond characterizations of individual cell properties, was critical to better understand how those signals are utilized and how they differ across cortical areas. Previous studies of eye-position modulations, including Xu et al. (2012) and Morris et al. (2012), have generally just reported the presence of such modulations in individual cells without analyzing the data with any form of population decoding (Andersen and Mountcastle, 1983; Andersen et al., 1985, 1990; Galletti and Battaglini, 1989; Ringo et al., 1994; Galletti et al., 1995; Squatrito and Maioli, 1996; Bremmer et al., 1997; Guo and Li, 1997; Boussaoud et al., 1998; Trotter and Celebrini, 1999; Bremmer, 2000; DeSouza et al., 2002; Rosenbluth and Allman, 2002; Lehky et al., 2008).

### Method: advantages of intrinsic vs. extrinsic population decoding analyses

One recent study has subjected eye-position modulations to a population coding analysis (Morris et al., 2013) and also reported findings in disagreement with the conclusions of Xu et al. (2012) that eye position information was too unreliable to be used in real time (see also Kaplan and Snyder, 2012). Consistent with our findings, Morris et al. (2013) report that eye positions and stimulus locations can be decoded from short-duration dorsal stream eye position signals. Although Morris et al. (2013) included a population analysis of eye-position data, their data were confined to dorsal stream cortical areas, namely LIP, VIP, and MT+ (i.e., MT and MST), without data from the ventral stream. A second key methodological difference between our study and that of the Morris et al. (2013) is that we adopted an intrinsic method, MDS, for our population decoding analysis while they used extrinsic methods, Bayesian classification and maximum likelihood estimation. A consequence of these different approaches to analyzing the data is that they had to label each neuron with information about its gain field whereas we did not.

#### No need for labeling

We refer to MDS as an *intrinsic* decoding method, because it is based purely on neural firing rates without any additional information about the receptive field characteristics of neurons in the population. Because there is no additional information besides firing rate, the neurons are unlabeled. In contrast, for *extrinsic* decoding methods such as weighted peak averaging or Bayesian estimation, neurons require labeling with additional information besides firing rate, such as the location of tuning curve peaks or response statistical distributions (for recent reviews of more common extrinsic approaches, see Pouget et al., 2000; Sanger, 2003; Averbeck et al., 2006; Quian Quiroga and Panzeri, 2009). As Morris et al. (2013) state, their analysis “used knowledge of individual eye-position tuning properties to estimate the associated eye position.” Differences between the extrinsic and intrinsic approaches are extensively discussed in our recent review on population coding (Lehky et al., 2013). In sum, an intrinsic approach, such as that used here, has advantages both in terms of physiological plausibility (no need for labeling) and representation (such as invariance).

#### Relational and invariant

Besides the labeled/unlabeled distinction, a second important distinction between intrinsic and extrinsic approaches is relational vs. atomistic representations. Intrinsic representations are relational while extrinsic representations are atomistic. With respect to the problem of coding eye positions, a relational representation means that the relative positions of a set of eye positions are extracted from neural activity, rather than the absolute positions of individual eye positions, which provides advantages with respect to invariances (for further discussion, see Lehky et al., 2013).

#### Greatest precision at small eccentricities

We calculated a local measure of spatial error, precision (Figure 5B), and found that spatial precision (standard error for individual eye positions) increased as gaze angle increased, both in dorsal and ventral areas. Therefore the greatest spatial precision for eye-positions occurred at small eccentricities. In contrast, Morris et al. (2013) found an opposite relation, with the least precise spatial representation (highest spatial error) at the central eye position, improving as eccentricity increased. Although we feel it unlikely, it is possible that there are sample differences between the studies, given the relatively limited populations of recorded cells in both studies. It remains for future experimental and theoretical investigations to separately examine eccentricity effects in detail and test how eye position signals influence perception and how spatial encoding is influenced by various receptive field properties, stimulus conditions, and/or analysis assumptions.

### Ventral vs. dorsal streams

The visual system is divided into a ventral stream extending into inferotemporal cortex, and a dorsal stream extending into structures in parietal cortex. This division received widespread attention following the work of Ungerleider and Mishkin (1982), who associated the two streams with different aspects of perceptual processing leading to the classic what/where dichotomy. Over the decades, various researchers built upon this fundamental distinction. Goodale and Milner (1992) proposed an influential revised account of the differences between ventral and dorsal processing, in which the previous perceptual what/where dichotomy became a perception/action dichotomy. Both Jeannerod and Jacob (2005) and Rizzolatti and Matelli (2003), while accepting the perception/action distinction, further elaborated on it and re-emphasized that the dorsal stream is not purely engaged in controlling action but does have a perceptual aspect as well. Recent work has shown that while shape (e.g., Sereno and Maunsell, 1998; Sereno et al., 2002; Konen and Kastner, 2008) and spatial (e.g., Sereno and Lehky, 2011) representations are present in both dorsal and ventral streams, they often differ from each other in significant ways (Lehky and Sereno, 2007; Sereno and Lehky, 2011).

Space is a fundamental aspect of visual representation both in the dorsal and ventral visual streams. In the dorsal stream, the location of an object is obviously important during visuomotor control of actions. In order to grasp or make a saccade to an object, its location must be represented. In the ventral stream, the spatial arrangement of different features of an object must be registered to form a coherent object percept (Edelman, 2002; Newell et al., 2005). The inability to synthesize an object percept from local features may underlie some forms of visual agnosia (Farah, 2004). Likewise, the locations and spatial relationships among multiple objects are important in scene perception (Hayworth et al., 2011) and cells modulated by changes in the spatial arrangement of stimuli have been reported in the ventral stream (e.g., Missal et al., 1999; Aggelopoulos and Rolls, 2005; Messinger et al., 2005; Yamane et al., 2006).

Although space is important in both dorsal and ventral streams, a widespread assumption is that any spatial information or spatially selective modulations of ventral areas are derived from inputs from dorsal stream areas (e.g., Ungerleider and Haxby, 1994; Corbetta and Shulman, 2002; Kravitz et al., 2011). In contrast, we have previously reported that there are dorsal-ventral differences in the representation of retinotopic space (Sereno and Lehky, 2011) and have argued that spatial information in the two streams, like shape information in the two streams (Lehky and Sereno, 2007), is developed in parallel and is largely independent.

Support for a minimum of two independent spatial representations in the visual system also comes from neuropsychological studies reporting implicit feature binding (without visual awareness) remaining in a patient with posterior parietal damage who lacks the more familiar explicit feature binding (Friedman-Hill et al., 1995; Wojciulik and Kanwisher, 1998). As binding requires location or spatial information, those results suggest two qualitatively distinct spatial representations, possibly in dorsal and ventral streams (Wojciulik and Kanwisher, 1998), as the authors of this study also suggest.

Likewise, based on neurophysiological findings, we have previously argued that the differences between dorsal and ventral streams is not based on a strict dichotomy between shape and spatial processing (Lehky and Sereno, 2007; Sereno and Lehky, 2011). Rather, each stream independently encodes both shape and spatial information, but with differences in the encoding of shape and spatial signals in each case, with each stream geared to different functionalities. For the dorsal stream areas, precise coordination of the body (hand, eye, etc.) position with respect to object or world position is critical for real-time control of action and navigation. Hence a metrically accurate (or *coordinate*) *egocentric* spatial reference frame is required. On the other hand, spatial representations for recognition and memory in the ventral stream are concerned with relations of objects (or parts of objects) with each other rather than with the body, so that they may more often operate within an *allocentric* reference frame. Oftentimes, it is sufficient for these ventral spatial representations to be *categorical* (i.e., to the left of, on top of, etc.) rather than *coordinate* (metrically accurate). Kosslyn et al. (1989); Kosslyn et al. (1992) discuss the distinction between categorical and coordinate spatial representations.

### Ventral vs. dorsal differences in eye position based spatial representations

In this study we have found dorsal/ventral differences in the encoding of eye-position space, extending previous work comparing dorsal/ventral encoding of shape (Lehky and Sereno, 2007) as well as dorsal/ventral encoding of retinotopic space (Sereno and Lehky, 2011). We found that while responses in AIT and LIP were both capable of representing spatial maps of eye position, the map formed by AIT was more distorted (comparing Figures 3B and C), with spatial distortion measured as stress [Equation (2)]. Nevertheless, the AIT map still retained the correct topological relationships. That a ventral structure such as AIT is capable of forming a representation of space purely on the basis of eye-position modulations is a significant departure from current thinking. The higher accuracy of LIP spatial representations and lower accuracy of AIT spatial representations for eye-position space we found here mirrors the same characteristics we previously reported for retinotopic space (Sereno and Lehky, 2011).

One possible interpretation for the observed differences in eye-position accuracy is that LIP, as a dorsal structure, is forming a metric or coordinate representation of space required for accurate visual control of motor actions, whereas AIT, as a ventral structure, is forming a categorical or qualitative representation of space sufficient for object categorization or organizing the contents of a scene. For example, if the ventral stream is processing a visual scene for input to memory, it may be sufficient to localize different objects in a qualitative manner, “to the left of… ” or “on top of… ” rather than in a metrically or numerically precise manner. While our results do lead to an interpretation of dorsal/ventral differences in terms of the metric/categorical distinction in spatial representations, the experimental design doesn't directly test the allocentric/egocentric distinction.

### Ventral vs. dorsal differences in spatial distortions across eccentricity and laterality

We found greater spatial distortion [i.e., stress, Equation (2)] as gaze angle increased in AIT but not LIP (Figure 5A), indicating less accurate representations of eye-position space at larger eccentricities for the ventral area. This is different from what we observed for retinotopic space, where spatial distortion increased with retinal target eccentricity for both AIT and LIP (Sereno and Lehky, 2011).

In considering this difference between eye-position space and retinotopic space, it should be noted that stimulus conditions are quite different in the two cases. For eye-position space as examined in this study, the stimuli are at fixation straddling both the ipsilateral and contralateral visual fields. It is only position of the eye in the orbit that is deviated to the ipsilateral or contralateral side. For retinotopic space, the visual stimuli were presented to the ipsilateral or contralateral visual field at some eccentricity away from fixation.

Given these differences, one possibility why there was no eccentricity-related degradation in the accuracy of eye-position representations in LIP, while such degradation occurred in AIT, is the following. If dorsal structures use visual information in the region near fixation to most accurately guide hand and arm movements, then it would be critical that space in the fixation region be represented in a metrically accurate manner for all gaze angles. On the other hand, ventral structures are not involved in controlling motor actions in the physical world, and space there may be represented in a more qualitative, categorical manner in which greater distortions at large gaze angles were tolerable. One consequence of such differences suggests that if ventral stream areas are important for allocentric representations, then allocentric spatial representations of an object or scene may be more spatially distorted for information encoded at larger gaze angles, and when such stimuli are encoded at ipsilateral angles of gaze.

On the other hand, for retinotopic space (Sereno and Lehky, 2011), increased spatial distortion away from fixation for both dorsal and ventral streams may simply reflect generally reduced accuracy requirements in the visual periphery. Modeling indicates that this increased retinotopic spatial distortion with eccentricity is not dependent on larger diameter RFs in the periphery, as increased distortion still occurs for models with constant RF diameter (Lehky and Sereno, 2011).

### Ventral vs. dorsal differences in onset of eye position signals

Another dorsal/ventral difference is apparent upon examining Figure 2B, plotting PSTHs at the best and worst eye positions. In LIP, eye-position modulation starts substantially before there is any change in eye position (before zero time in the plot, marking saccade to target). On the other hand, in AIT the start of the eye-position modulation comes much later, roughly coinciding with the start of the eye movement.

Previous work has reported predictive receptive field remapping in the dorsal stream, in which receptive fields shift before the eye actually moves (Duhamel et al., 1992; Colby and Duhamel, 1996; see also Nakamura and Colby, 2002). Based on timing considerations (reviewed by Wurtz and Sommer, 2004), receptive field remapping has been attributed to motor efference copy (i.e., corollary discharge signals associated with the motor plan), which can precede the eye movement, rather than a proprioceptive signal from change in muscle length, which must lag the eye movement. Our LIP data is not inconsistent with a motor efference copy source for the early eye-position modulation that we observed, as those eye-position modulations arose before the onset of the saccade.

An alternative interpretation for what appears to be LIP eye-position modulation preceding saccade onset in our data is that it could instead be attributed to variable locations of the target stimulus, which was present in the periphery during that period. The target appears about 200 ms before the eye movement to target, with the target placed at various retinotopic locations in different trials. Modulations before the eye movement could have been due to visual responses to that target or some variable associated with those responses, and not eye-position modulations. However, we don't think that is the case. As we reported above, eye-positions producing strong responses are uncorrelated with retinotopic positions producing strong responses in both areas. The two plots in each panel of Figure 2B were selected for “best eye position” and “worst eye position” responses. That means any retinal responses contaminating our signal, being uncorrelated with eye position, would be randomly assigned to the “best eye position” and “worst eye position” plots. Therefore, differences due to retinal stimulation would be averaged away.

It is difficult for us to give a definitive interpretation as to why eye-position modulations occur earlier in LIP than AIT, as this study was not designed to be able to distinguish the source of the eye position signals. Wurtz and Sommer (2004) review and prescribe several criteria necessary to identify corollary discharge signals in the primate brain, criteria that the current study cannot address. Nevertheless, one possible interpretation is that eye-position modulation in LIP depends on motor efference (corollary motor discharges), whereas eye-position modulation in AIT, where significant modulation starts shortly after the saccade onset (35 ms post-saccade, Lehky et al., 2008), depends on proprioceptive signals. A second, perhaps more likely, possibility, is that AIT modulations as well as LIP modulations have their source in corollary motor discharges. Eye-position modulations due to corollary motor discharges do not necessarily precede eye movements, but can coincide or lag them as well, depending on the latency with which the motor efference copy signal reaches visual cortex (Wurtz and Sommer, 2004). Therefore it may be that eye-position modulations in LIP and AIT both depend on motor efference copy, but the efference copy signal has a longer latency in AIT.

Interestingly, both areas show an additional marked change in eye-position signals occurring with a lag after saccade onset with LIP again showing a shorter lag (~40 ms after saccade onset) than AIT (~80 ms after saccade onset). First, this suggests the possibility that there may be multiple sources of eye-position information within each stream, one slower than the other, possibly reflecting different contributions from both motor efference copy and proprioception. Second, the longer lags that occur for not only the initial but also the later AIT eye-position modulation may indicate longer latencies in general for eye-position signals to reach AIT. Differences in eye-position signal timings between streams may therefore reflect feedforward or feedback conduction delays specific to each stream due either to differences in the number of processing stages within each stream or where or when the eye-position signal has entered each stream (i.e., different stages in hierarchy). It is noteworthy that AIT has longer latencies for both eye-position signals and visual responses, and any connection between the two latencies remains to be investigated.

The dynamics of eye-position modulations in the ventral stream have not been investigated other than in this report and our own previous report on AIT (Lehky et al., 2008). The dynamics of eye position signals, the role of corollary discharge and proprioception, and presence/absence of remapping should be examined on AIT cell responses using the same techniques and criteria developed and established in superior colliculus, frontal eye field, and other dorsal stream areas. Such studies, which have never been done in ventral stream areas, would be able to elucidate the source of those modulations (proprioceptive feedback or corollary discharges) and perhaps be able to account for the later occurrence of the onset of eye-position modulations in AIT. The comparative dynamics of eye-position modulations in the two streams merits further examination.

### Direct decoding of eye position modulations vs. coordinate transforms

A major focus of current theoretical thinking on the role of eye position modulations is that they may be involved in a transform of visual spatial coordinates from an eye-centered reference frame to a head-centered reference frame. This idea was introduced by Andersen et al. (1985) and formalized in the neural network model of Zipser and Andersen (1988). In the years since, it has been elaborated and applied to the interpretation of various neural data sets (Andersen et al., 1993; Pouget and Sejnowski, 1997; Boussaoud and Bremmer, 1999; Pouget and Snyder, 2000; Snyder, 2000; Salinas and Abbott, 2001; Lehky et al., 2008).

The approach taken here fundamentally differs from the above in that eye position modulations are used to directly decode gaze angle, and not to transform retinotopic spatial coordinates. There are no coordinate transforms of visual space in our analysis of eye-position modulation data.

The variable we were directly extracting is eye position, which is equivalent to the *position of a stimulus* at fixation. Finding stimulus location in this manner does not require performing any coordinate transformations. Successively fixating different objects can determine the relative positions of multiple stimuli. Such scanning of the visual field typically occurs at a rate of 3–5 saccades/s (Rayner and Pollatsek, 1992; Rayner, 1998, 2009). Building up relative spatial maps of stimuli across fixations may be similar to active vision approaches (Ballard, 1991; Findlay, 1998) for constructing representations of space using eye movements.

Although we demonstrate the ability to extract stimulus target location with eye-position signals alone, we are not arguing that spatial representations in a given area must be limited to a single spatial input signal. It is highly likely that different sources of spatial information (e.g., eye angle, head angle, or vestibular signals), including information ultimately based on retinotopic coding, may be combined in different ways at different levels in the system. Therefore, recovering object locations using changes in eye position supplements rather than supersedes current ideas based on computing a series of spatial coordinate transforms (e.g., Andersen et al., 1993). Nevertheless, we show here that spatial representation need not proceed by coordinate transformations, and that eye position spatial information alone contained within small populations of cells in both a ventral and dorsal area are sufficient to fairly accurately recover target stimulus location.

### Modeling eye position based representations

We present a simple population-coding model of eye-position representation based on gain fields. Decoding was based on applying MDS to synthetic data from model neurons, in a manner identical to the way MDS was applied to actual data. The success of the model in finding that eye position can be directly decoded from a population of neurons having a variety of gain fields highlights several important issues: (1) modeling success with decoding eye position signals supports our physiological findings that eye position can be directly decoded from a population of neurons; (2) modeling success also demonstrates that recovering space from eye-position modulations is not an artifact of the data or data analysis methods; (3) modeling is an indication that we have an understanding of the processes that generated our data; and (4) having a model opens the way for future modeling studies, under well-controlled parametric conditions, examining how gain field characteristics affect the representation of eye-position space.

In addition, we show that MDS modeling can fail to recover visual space for particular gain field characteristics (i.e., when all the sigmoidal gain fields in the population were close to anti-symmetric around the central eye position). This failure demonstrates that our approach is not tautological. It is possible that other configurations of gain fields may also fail to completely recover spatial information, a topic for future modeling studies. Such failures point out the advantages of using modeling, which may highlight aspects of receptive field characteristics and organization whose significance might otherwise not be apparent.

Further development of gain field models of eye position may help us to understand how differences in gain field characteristics in different cortical areas lead to differences in spatial representations in those areas, as well as how individual parameters describing gain fields affect spatial representation. Although many cortical areas show eye-position modulations, quantitative modeling might also be helpful for identifying cortical areas with characteristics that optimize the recovery of eye-position information for particular applications. Such information could be useful for guiding the development of brain-computer interfaces in neuroprosthetic systems.

## Conclusion

In sum, spatial representations are not confined to the dorsal stream. We unequivocally show here, in a first study to use population-decoding methods to analyze eye-position modulations across visual streams, that visual space is represented in both dorsal and ventral structures. However, more detailed examination of how physiological eye position signals in the two streams differ and how those differences affect population decoding of those signals and relate to functional differences between the streams is needed. The nature of the eye position based spatial representation in the two streams is an unexplored frontier that, with the help of novel intrinsic population decoding techniques, promises to change our understanding of cortical spatial representation.

## Author contributions

Anne B. Sereno conceived and designed the experiments, collected the data, and helped with data analysis and modeling. Sidney R. Lehky did the data analysis and modeling. Anne B. Sereno, Margaret E. Sereno, and Sidney R. Lehky wrote the manuscript.

### Conflict of interest statement

The authors declare that the research was conducted in the absence of any commercial or financial relationships that could be construed as a potential conflict of interest.
